# Cystatin C and asymptomatic coronary artery disease in patients with metabolic syndrome and normal glomerular filtration rate

**DOI:** 10.1186/1475-2840-11-108

**Published:** 2012-09-14

**Authors:** Xie Qing, Wang Furong, Liu Yunxia, Zhang Jian, Wang Xuping, Gao Ling

**Affiliations:** 1 Department of Central Laboratory, Provincial Hospital affiliated to Shandong University, Jinan, People’s Republic of China; 2 Department of Pharmacy, Qilu Hospital of Shandong University, Jinan, People’s Republic of China; 3 Institute of Pharmacology, School of Medicine Shandong University, Jinan, People’s Republic of China; 4 Department of Pharmacology, Shandong University of Traditional Chinese Medicine, Jinan, People’s Republic of China; 5 Department of Epidemiology and Health Statistics, School of Public Health, Shandong University, Jinan, People’s Republic of China; 6 Department of Clinical Laboratory, Qilu Hospital of Shandong University, Jinan, People’s Republic of China; 7 The Key Laboratory of Cardiovascular Remodeling and Function Research, Chinese Ministry of Education and Chinese Ministry of Health, Qilu Hospital of Shandong University, Jinan, People’s Republic of China

**Keywords:** Cystatin C, Gensini score, Metabolic syndrome, Asymptomatic coronary artery disease

## Abstract

**Background:**

All of the components of Metabolic syndrome (MetS) have been regarded as risk factors for coronary artery disease (CAD). Early detection of CAD in asymptomatic patients with MetS remains a challenge. Cystatin C,which has been proposed as a novel marker of renal dysfunction,is correlated with mortality in CAD, The purpose of the study was to evaluate whether cystatin C is a potential marker of asymptomatic CAD in MetS patients with normal kidney function.

**Methods:**

A total of 211asymptomatic MetS patients without prior history of CAD patients were included in a cross-sectional study. Patients were divided into MetS with asymptomatic CAD (n = 136) and MetS without CAD (n = 75) groups according to coronary angiograph results. Serum cystatin C levels were measured using particle enhanced immunonephelometric assays. We first assessed whether there is an independent association of cystatin C with the presence and severity of asymptomatic CAD. Then, we investigated the association between cystatin C and other biochemical risk factors for atherosclerosis.

**Results:**

Serum cystatin C levels in patients with asymptomatic CAD were significantly higher than those without CAD (*P =* 0.004). A multiple logistic regression analysis demonstrated cystatin C was independently associated with the presence of asymptomatic CAD (OR = 1.326, 95%CI: 1.086-1.619). On receiver operating characteristics (ROC) analysis, the area under the curve (AUC) was 0.622 (95 % CI: 0543–0.701, *P* = 0.003), and cystatin C showed a moderate predictive value. Furthermore, cystatin C was independently correlated with Gensini score (standardized β = 0.183, *P* = 0.007), and serum cystatin C levels increased with the increasing of number of disease vessels (P = 0.005). In a multiple stepwise regression analysis, uric acid (UA)(*P* < 0.001), body mass index (BMI)(*P =* 0.002), triglyceride(TG)(*P* = 0.03), estimated glomerular filtration rate (eGFR)(*P* < 0.001), and fibrinogen*(P* = 0.001) were independently associated with cystatin C.

**Conclusions:**

Serum cystatin C in our study was significantly associated with the presence and severity of asymptomatic CAD in MetS patients with normal kidney function, suggesting that cystatin C is probably more than a marker of glomerular filtration rate.

## Background

Metabolic syndrome (MetS) is a clinical entity characterized by abdominal obesity, hyperglycaemia, hypertension, and dyslipidaemia. Until now, the pathophysiology of MetS is not well defined [1]. Each of the components have been recognized as risk factors for coronary artery disease (CAD)[2], and today a series of previous studies have demonstrated that the presence of MetS is associated with an increased risk of developing CAD [3,4]. CAD is the leading cause of morbidity and mortality worldwide. Asymptomatic patients have a higher cardiac mortality risk than those with symptomatic CAD [5]. An early identification and treatment of asymptomatic CAD patients may significantly improve their cardiovascular prognosis. Unfortunately, the early diagnosis of asymptomatic CAD is always missed or delayed because the typical symptoms of cardiac ischemia are often masked. To date, classic assessment such as Framingham Risk Score (FRS) could not identify asymptomatic CAD effectively. Biochemical markers might play crucial roles on initial assessment of asymptomatic CAD.

Cystatin C belongs to a family of competitive inhibitors of lysosomal cysteine protease and is synthesized at a constant rate in all nucleated cells [6]. Compared with serum creatinine, cystatin C is less influenced by age, sex, and race, and a combined creatinine-cystatin C equation performed better than equations that used creatinine alone for classification of the estimating glomerular filtration rate (eGFR) [7]. Cystatin C has emerged as a novel sensitive marker for detecting renal dysfunction [8,9]. Recent studies have revealed that cystatin C is not simply regarded as a candidate marker of impaired kidney function. In Prospective Epidemiological Study of Myocardial Infarction (PRIME), cystatin C predicted the occurrence of the first coronary events in men aged 50 to 59 years old, and displayed a strong relation with CAD independent of eGFR [10]. Several other publications have demonstrated that cystatin C was closely associated with incident congestive heart failure [11], carotid atherosclerosis [12] and peripheral vascular disease[13] superior to serum creatinine or creatinine-based eGFR.

In addition to the association with atherosclerosis, connections between cystatin C and features of the MetS have also been illustrated by a few recently published studies [14,15]. In a cross-sectional study included 925 dyslipidaemic patients, Aude Servais et al. [16] found that a progressive increase in serum cystatin C occurred in parallel with an increase in the number of MetS components. To date, the link between cystatin C and asymptomatic CAD in MetS patients remains to be elucidated. The current study was designed to investigate whether there is an independent association of cystatin C with the presence and severity of asymptomatic CAD in MetS patients with normal kidney function and to explore the relationship between cystatin C and other biochemical risk factors for atherosclerosis.

## Methods

### Study subjects

This was a cross-sectional study consisted of 211 asymptomatic MetS patients without prior diagnosed CAD and with normal eGFR who presented for a annual medical evaluation at QiLu Hospital of Shandong University (Jinan, China)from March 2010 to March 2012. All patients had abnormal ECG findings include the ST segment abnormalities, T wave abnormalities, and other abnormalities indicate myocardium ischemia and referred to a coronary artery angiography for suspected asymptomatic CAD. The patients were divided into MetS with asymptomatic CAD (n = 136) and MetS without asymptomatic CAD (n = 75) groups according to their coronary angiography results. Individuals with angina pectoris or anginal-equivalent symptoms suggestive of CAD, valvular heart disease, malignancy, inflammatory disease, renal dysfunction (creatinine-based eGFR <90 mL/min/1.73 m^2^ calculated by Cockcroft-Gault formula, serum creatinine > 133umol/L), hepatic dysfunction were excluded from the study. Studies have shown that large doses of glucocorticoids would increase the production of cystatin C [17]. Thyroid dysfunction also has a major impact on cystatin C levels. Serum levels of cystatin C are lower in the hypothyroid state and higher in the hyperthyroid state [18,19]. Patients with above factors were also excluded from this study to eliminate potential confounding factors. The study protocol complied with the Declaration of Helsinki and was approved by the Clinical Research Ethics Committee of QiLu Hospital, Medical College of Shandong University. Written informed consent was obtained from all subjects.

### Diagnostic criteria

(1)The diagnostic criteria for MetS were based on the revised Adult Treatment Panel III criteria (revised ATP-III) [20]. Because waist circumference was not available in this study, BMI was employed as a surrogate for waist circumference as has been used to define MetS by the Chinese Diabetes Society [21]. Patients met with at least three or more of the following conditions were diagnosed with MetS :

Reduced high-density lipoprotein cholesterol (HDL-C): ①HDL-C <1.03 mmol/L (male) or < 1.29 mmol/L (female); ②Hyperglycaemia: fasting plasma glucose (FPG) ≥ 5.6 mmol/L or antidiabetic medication use; ③Hypertension: systolic blood pressure or diastolic blood pressure(SBP/DBP) ≥ 130/85 mmHg and/or diagnosed hypertension treated with antihypertensive therapy; ④Elevated triglycerides (TG): TG ≥ 1.7 mmol/L or receiving specific treatment for this lipid abnormality; ⑤Body mass index (BMI) ≥25 kg/m^2^.

(2)Asymptomatic CAD patients were diagnosed according to the Monica criteria [22]: two or more ECG showing specific changes without suggestive symptoms, and confirmed by the presence of at least one lesion with ≥50% luminal stenosis by coronary angiography. Abnormal ECG findings include the ST segment abnormalities, T wave abnormalities, and other abnormalities indicate myocardium ischemia.

### Clinical and biochemical assessment

Demographic characteristics and cardiovascular risk factors were obtained from medical records. The cardiovascular risk was assessed in terms of hypertension (blood pressure ≥140/90 mmHg or taking anti-hypertensive drugs), smoking status (≥20 packs per year), and diabetes mellitus (fasting plasma glucose level ≥ 6.1 mmol/L or taking insulin or oral hypoglycaemic drugs). Hypercholesterolemia (total cholesterol level ≥5.0 mmol/L or use of lipid-lowering medication). BMI was calculated as weight (kg) divided by the square of the height (m^2^). Systolic and diastolic blood pressures were measured using standard methods.

Venous blood was collected after overnight fasting before coronary artery angiography. Serum was stored at −80°C and was used to analyse of total cholesterol (TC), low-density lipoprotein cholesterol (LDL-c), high-density lipoprotein cholesterol(HDL-c), triglycerides (TG), uric acid(UA) and fasting plasma glucose(FPG) using an automatic biochemistry analyzer (Roche Cobas 8000 modular analyzer Series C701, Mannheim, Germany). Concentrations of serum cystatin C were determined using Roche Cobas 8000 modular analyzer Series (Roche, Inc., Mannheim, Germany) with a particle-enhanced immunonephelometric assay (Roche Tina-quant Cystatin C, Roche, Inc., Mannheim, Germany).The laboratory reference ranges for cystatin C was 0.4 to 8.0 mg/L, and the intra-assay and inter-assay coefficients of variation were 0.9 % and 2.9 %, respectively. Fibrinogen was measured using Von Clauss methods on an electromechanical coagulometers (STA-R, Diagnostica Stago, Asnieres, France). Serum creatinine(sCr) levels were measured using an enzymatic method, and eGFR was estimated with the Cockcroft-Gault formula [23]. Participants with an eGFR < 90 mL/min/1.73 m^2^ were excluded.

### Coronary angiography

Coronary artery angiography was performed using standard Judkins techniques. Angiographic analysis was conducted by two expert investigators who were blinded to the study protocol. The extent of CAD was described as 0-, 1-, 2-, or 3-vessel disease based on the number of coronary vessels with >50% luminal narrowing. The severity of coronary atherosclerosis was scored based on Gensini scoring [24].

### Statistical analysis

Continuous variables with normal distribution were expressed as the mean ± SD, while percentages were used to express categorical variables. Variables such as TG, HDL-C, Gensini score that were not normally distributed, as determined by the Kolmogorov–Smirnov test, were logarithmically transformed before analysis and expressed as medians with inter quartile range. The chi-square test was used to compare the categorical variables of subjects with and without CAD. An unpaired student’s *t* test was used for normally distributed variables and the Mann–Whitney *U*-test was used for skewed variables. Pearson and partial correlation analysis were applied to determine factors that correlated with cystatin C, and Spearman correlation analysis was performed to determine factors that correlated with Gensini score. To determine the independent parameters correlated with cystatin C and the Gensini score, the parameters that correlated significantly in the univariate analysis and other parameters that were biologically relevant to cystatin C and the Gensini score were tested using multiple stepwise regression analysis. A logistic regression analysis was performed to assess the association between the presence of CAD and cystatin C levels. Age, gender, BMI, smoking state, hypertension, diabetes, lipid profile(TC,TG LDL-c, HDL-c), and eGFR were considered to be potential confounders and were adjusted in the different models. In addition, the receiver operating characteristics (ROC) curve was used to determine the cystatin C cut-off for predicting asymptomatic CAD. All statistical analyses were performed using SPSS 16.0 software for Windows (SPSS, Chicago, IL, USA). A two-tailed P-value < 0.05 was set as the level of significance.

## Results

### Characteristics of the study subjects

**Figure 1:**
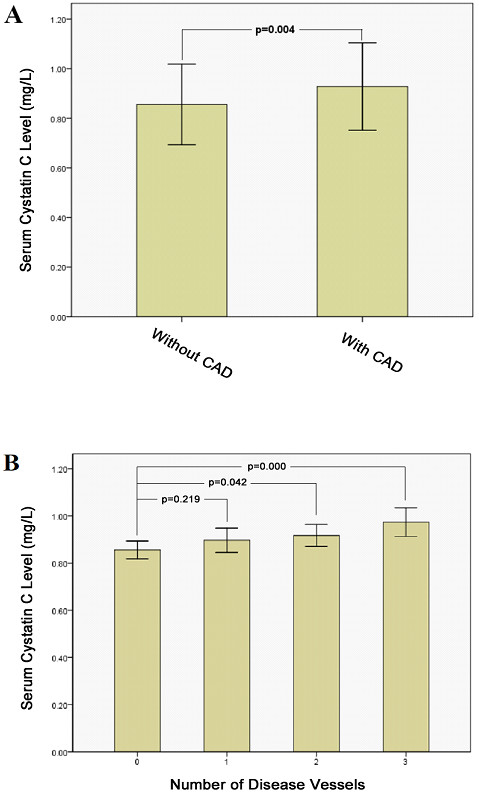
**Serum cystatin C levels between MetS patients with asymptomatic CAD (n = 136) and those without CAD (n = 75) (A).** CAD, coronary artery disease; MetS, metabolic syndrome. Comparison of serum cystatin C levels according to the number of stenotic coronary arteries (n = 75, 40, 56, 40, respectively, in 0, 1, 2and 3 stenotic vessels group)(**B**).

The characteristics of the study subjects are described in Table 1. The patients with asymptomatic CAD were older than those without CAD(*P* = 0.011).The rates of hypertension and diabetes mellitus in patients with asymptomatic CAD were higher than in patients without CAD, and asymptomatic CAD patients showed an increase of FPG (*P* = 0.011). The fibrinogen concentrations were significantly higher in patients with asymptomatic CAD than in those without CAD (*P* = 0.012). However, there was no difference in sCr, eGFR and lipid profiles between patients with asymptomatic CAD and without CAD. Among the patients with MetS, those with asymptomatic CAD had significantly higher serum cystatin C levels than those without CAD (0.93 ± 0.18 vs 0.86 ± 0.16 mg/L, *P* = 0.004; Figure 1A).

**Table 1:** Clinical and biochemical characteristics of the study subjects

	**Non-CAD(n = 75)**	**CAD(n = 136)**	***P***
Age(years)	55.05 ± 9.47	58.82 ± 10.54	0.011
Males% (n)	57(43)	65(88)	0.291
Current smokers%(n)	31(23)	46(74)	0.027
Hypertension%(n)	68(51)	78(106)	0.113
Diabetes%(n)	33(25)	37(50)	0.618
BMI(kg/m^2^)	27.43 ± 2.20	27.24 ± 2.57	0.580
Systolic BP(mmHg)	141.11 ± 17.05	141.79 ± 19.05	0.801
Diastolic BP(mmHg)	82.08 ± 13.33	81.161 ± 14.29	0.648
HR(bpm)	74.67 ± 12.66	72.82 ± 11.69	0.289
FPG(mmol/L)	5.97 ± 1.34	6.53 ± 1.58	0.011
TC(mmol/L)	4.86 ± 1.12	5.02 ± 1.14	0.341
TG(mmol/L)	1.92(1.58-2.18)	2.09(1.48-2.57)	0.197
LDL-c(mmol/L)	2.82 ± 0.85	2.89 ± 0.93	0.549
HDL-c(mmol/L)	1.06(0.93-1.23)	1.06(0.91-1.21)	0.541
SCr(mmol/L)	70.73 ± 16.74	70.56 ± 13.59	0.938
CG-eGFR(ml/min/1.73 m^2^)	112.04 ± 27.60	104.92 ± 26.04	0.064
UA(μmol/L)	319.00 ± 82.47	331.56 ± 94.53	0.335
Fibrinogen(g/L)	3.30 ± 0.68	3.58 ± 0.78	0.012
Cystatin C(mg/L)	0.86 ± 0.16	0.93 ± 0.18	0.004
Gensini score	8.00(2.5-12.00)	42.0(27–53.75)	<0.001

BMI, body mass index; BP, blood pressure; HR, heart rate; FPG, fasting plasma glucose; TC, total cholesterol; TG, triglyceride; LDL-c, low-density lipoprotein cholesterol; HDL-c, high-density lipoprotein cholesterol; SCr, serum creatinine; UA, uric acid. Data are means ± SD or median (interquartile range).

### Relationship between serum cystatin C and the presence of asymptomatic CAD

Multiple logistic regression analysis was performed using the presence of the asymptomatic CAD as a dependent variable (Table 2). The analysis involved age, sex, BMI, smoking, hypertension, diabetes and biochemical risk factors, including TC LDL-c, HDL-c, TG, SBP, DBP, fibrinogen, FPG, eGFR and cystatin C. As a result, serum cystatin C (odds ratio, OR = 1.326, 95% confidence interval(CI):1.086-1.619, *P* = 0.006), fibrinogen (OR = 1.629, 95% CI: 1.043-2.543, *P* = 0.032), FPG (OR = 1.363, 95% CI: 1.088-1.707, *P* = 0.007) and smoking (OR = 1.913, 95% CI: 1.007-3.633, *P* = 0.048) were independent predictors of the presence of the asymptomatic CAD.

**Table 2:** Multiple stepwise logistic regression analysis indicating factors independently associated with asymptomatic CAD

**Parameters**	**Exp(B)**	**95% CI**	***P***
Fibrinogen	1.629	1.043-2.543	0.032
FPG	1.363	1.088-1.707	0.007
Smoking	1.913	1.007-3.633	0.048
Cystatin C	1.326	1.086-1.619	0.006

CAD, coronary artery disease; CI, confidence interval; FPG, fasting plasma glucose.

Variables included in the model are age, sex, BMI, smoking, hypertension, diabetes and biochemical risk factors TC, LDL-c, HDL-c, TG, SBP, DBP, fibrinogen, FPG, eGFR and cystatin C.

**Figure 2:**
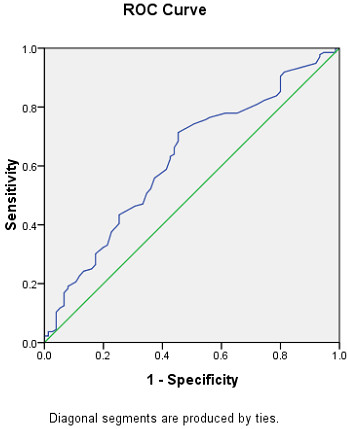
ROC curve analyses for predictive values of serum cystatin C levels in detecting asymptomatic CAD;ROC, receiver operating characteristic; CAD, coronary artery disease.

The ability of serum cystatin C levels to distinguish MetS patients with asymptomatic CAD from those without CAD was assessed using ROC curve analysis. The ROC curves for asymptomatic CAD diagnosis had an area under the curve (AUC) of 0.622 (95% CI: 0543–0.701, *P* = 0.003). A serum cystatin C level of ≥0.825 mg/L predicted the presence of CAD with a sensitivity of 71.3% and specificity of 54.7% (Figure 2).

### Correlation between serum cystatin C and the clinical biochemical parameters

We next investigated the relationship between serum cystatin C levels and clinical biochemical parameters. The Pearson correlation analysis demonstrated that serum cystatin C was positively correlated with age, UA, sCr, and fibrinogen and negatively correlated with eGFR (*P* < 0.01). After further adjustment for age, sex and eGFR, the positive correlation remained significant between cystatin C and UA, fibrinogen (*P* < 0.001, *P* = 0.001, respectively). A positive correlation was also observed between cystatin C and TG and BMI (*P* = 0.031, *P* < 0.001, respectively, Table 3). Furthermore, multivariate stepwise regression analysis was performed to evaluate the independent factors of cystatin C with age and the factors identified in the above univariate analysis including UA, BMI, TG, eGFR, and fibrinogen as independent variables. The analysis demonstrated that UA (standardized β = 0.233, *P* < 0.001), BMI (standardized β = 0.176, *P* = 0.002), TG (standardized β = 0.117, *P* = 0.03), eGFR (standardized β = −0.455, *P* < 0.001), and fibrinogen (standardized β = 0.187, *P* = 0.001) were independently associated with serum cystatin C.

**Table 3:** Correlation of serum cystatin C levels with clinical and biological parameters

**Variables**	**Cystatin C**		**Cystatin C (adjust for age sex and eGFR)**	
	**r**	**P**	**r**	***P***
Age(years)	0.267	<0.001	_	_
BMI(kg/m^2^)	0.062	0.379	0.299	<0.001
HR(bpm)	−0.091	0.193	0.002	0.981
Systolic BP(mmHg)	0.003	0.961	0.036	0.609
Diastolic BP(mmHg)	0.009	0.895	0.069	0.331
TC(mmol/L)	0.105	0.135	0.122	0.083
TG(mmol/L)*	0.048	0.495	0.152	0.031
LDL-c(mmol/L)	−0.004	0.956	0.006	0.930
HDL-c(mmol/L)*	0.007	0.925	−0.026	0.713
Fibrinogen(g/L)	0.173	0.013	0.242	0.001
FPG(mmol/L)	−0.053	0.455	−0.102	0.149
UA(μmol/L)	0.382	<0.001	0.370	<0.001
SCr(mmol/L)	0.464	<0.001	_	_
eGFR(ml/min/1.73 m^2^)	−0.473	<0.001	_	_

BMI, body mass index; HR, heart rate; BP, blood pressure; TC, total cholesterol; TG, triglyceride; LDL-c, low-density lipoprotein cholesterol; HDL-c, high-density lipoprotein cholesterol; FPG, fasting plasma glucose; UA, uric acid; SCr, serum creatinine.

*Log transformed before analysis.

### Correlation between serum cystatin C and CAD severity

**Figure 3:**
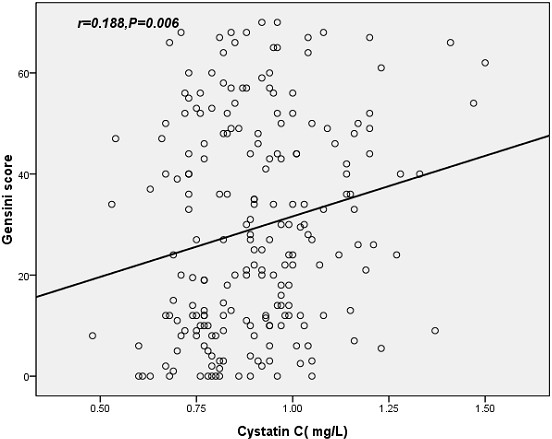
**Correlation between serum cystatin C and the severity of asymptomatic CAD evaluated by Gensini score.** CAD, coronary artery disease.

Spearman correlation analysis showed that serum cystatin C (r = 0.188, *P* = 0.006; Figure 3) was positively correlated with the Gensini score. We further performed a multivariate stepwise regression analysis to evaluate the association between serum cystatin C levels and the Gensini score in a model including age, sex, BMI, smoking, hypertension, and diabetes as well as TC LDL-c, HDL-c, TG SBP, DBP, fibrinogen, FPG, eGFR and cystatin C. The adjustment for the covariates did not change the associations significantly. Serum cystatin C was the independent factor that significantly influenced the Gensini score (standardized β = 0.183, *P* = 0.007, 95%CI: 0.602-3.844, Table 4).

**Table 4:** Multiple stepwise regression analysis showing variables independently associated with Gensini score

**Variables**	**Beta Coefficients**	***P***	**95% CI**
Cystatin C	0.183	0.007	0.602-3.844
FPG	0.195	0.004	0.867-4.483
Fibrinogen	0.184	0.007	1.418-8.856

FPG, fasting plasma glucose, CI, confidence interval.

Variables included in the model are age, sex, BMI, smoking, hypertension, and diabetes and biochemical risk factors TC LDL-c, HDL-c, TG SBP, DBP, fibrinogen, FPG, eGFR and cystatin C.

All of the subjects were further divided into four groups according to the number of disease vessels (N = 0, 1, 2, 3). The serum cystatin C levels were 0.86 ± 0.17, 0.90 ± 0.16, 0.92 ± 0.17 and 0.97 ± 0.19 mg/L in the groups with disease vessels of 0, 1, 2, and 3, respectively, and the serum levels of cystatin C increased as the number of stenotic vessels increased (*P* = 0.005).The cystatin C level in the group N = 0 was significantly lower than those in the groups of N = 2 and N = 3 (*P* = 0.042 and *P* ≤ 0.001, respectively, Figure 1B). No significance was found for cystatin C levels in the N = 0 and N = 1 groups. Moreover, the serum cystatin C levels were significantly higher in the N = 3 group than in the N = 1 group (*P* = 0.045).

## Discussion

In the present study, we demonstrated that in asymptomatic MetS patients without prior diagnosed CAD and with normal creatinine-based eGFR, elevated serum cystatin C was independently associated with the presence of asymptomatic CAD, even in an adjusted model. Serum cystatin C levels were positively and significantly correlated with the Gensini score. Furthermore, a positive association was also illustrated between serum cystatin C concentrations and the number of coronary arteries with luminal stenosis vessels. To the best of our knowledge, the study is the first to identify cystatin C as an independent risk factor for the development and severity of asymptomatic CAD in subjects with MetS and normal creatinine-based eGFR.

It is well recognized that patients with impaired renal function are at significantly higher risk for cardiovascular disease, congestive heart failure, all-cause mortality and adverse long-term outcomes in contrast to patients without renal disease [25-27]. Cystatin C has been regarded as a novel sensitive marker for the assessment of renal function, and the role of cystatin C as a predictor of cardiovascular events in patients with impaired renal function has been confirmed in clinical studies. In this study we assessed the association between cystatin C and asymptomatic CAD in a consecutive series of MetS patients with normal kidney function in order to avoid the well-known effect of overt renal insufficiency on coronary atherosclerosis, and evaluate whether cystatin C has an ability to identify individuals at a higher risk for CAD among patients belonging to a low-risk category according to eGFR. The current study demonstrated that serum levels of cystatin C, but not sCr and eGFR, were independently associated with the development of asymptomatic CAD, even after a variety of potential confounders were controlled. In accordance with our study, Wang et al. demonstrated elevated cystatin C was associated with the presence of CAD in subjects with mild renal impairment, while creatinine and eGFR were not able to predict CAD occurrence [28]. Similar findings were also observed by Koenig and his colleagues [29], who reported that cystatin C was superior to creatinine or eGFR for predicting cardiovascular events in a cohort of 1033 patients with CAD. Recently, the close relationship between cystatin C and all-cause cardiovascular mortality has been illustrated in subjects with normal eGFR [30], which further confirmed that cystatin C may not simply be regarded as an indicator of the association between renal dysfunction and an increased risk of CAD, the information contained by cystatin C represents more than just a marker of renal function.

Although cystatin C was independently associated with the presence of asymptomatic CAD,in the ROC analysis, the AUC for cystatin C falls into the range 0.6–0.7, which indicates the predictive value of cystatin as a sole marker was only moderate, and the cut-off value of 0.825 mg/L can not be categorized as ideal value to identify asymptomatic CAD effectively. Nevertheless, the addition of cystatin C may compensate for the inadequacy of. other classical parameters such as FRS for asymptomatic CAD. Future studies should investigate the feasibility of combined use of cystatin C and other parameters to improve the predictive value the presence of asymptomatic CAD.

In addition, when the associations between cystatin C and severity of asymptomatic CAD were evaluated, we found a positive relationship between serum levels of cystatin C and the number of diseased vessels. A positive association was also found between serum cystatin C levels and the Gensini score independent of eGFR, even after adjustment for established risk factors associated with cardiovascular disease and cystatin C. Similar results were also reported by Niccoli et al. [31], who demonstrated that the independent association between cystatin C and CAD severity was superior to that of creatinine or eGFR. In contrast, Kim et al. [32] failed to demonstrate an association between serum cystatin C and CAD in a retrospective study which included 64 diabetic patients, although they found an association between cystatin C and renal dysfunction. The small sample size might explain these discrepancies.

Detailed mechanisms underlying link between cystatin C and asymptomatic CAD have not been fully elucidated. Renal mechanism seems to be a plausible link between increased cystatin C and asymptomatic CAD. Serum cystatin C was assumed to be a sensitive indicator of“pre-clinical” renal disease which can not be detected by eGFR based on serum creatinine [33]. Cystatin C may thus help to identify individuals who are at increased risk for the development of asymptomatic CAD among patients with MetS and normal creatinine-based eGFR.

Besides renal mechanism, another possible explanation for the close relationship between cystatin C and. asymptomatic CAD is that cystatin C is associated with inflammation regardless of renal function Inflammation is a critical step in the development of atherosclerosis. Inflammatory cytokines associated with atherosclerosis may alter the relationship between cysteine protease and their endogenous inhibitors like cystatin C. The imbalance of cysteine protease and its inhibitor may increase the degradation of extracellular matrix and migration of monocytes, macrophages and vascular smooth muscle cells into the intima, thus leading to the development of atherosclerosis [34,35]. In the Heart and Soul Study, data from 990 patients with CAD indicated significant associations between cystatin C and proinflammatory parameters like C-reactive protein (CRP) or fibrinogen [36]. Similar results were shown in the Cardiovascular Health Study, which demonstrated cystatin C was correlated with CRP and fibrinogen in patients with mild to moderate renal dysfunction [37]. Consistent with these studies, we found a significant correlation between cystatin C and fibrinogen, whereas the correlations between fibrinogen and other renal markers such as serum Cr and eGFR were weak and insignificant. This may at least partly explain the complicated underlying link between cystatin C and asymptomatic CAD.

Uric acid has been shown to be related with increased production of oxygen free radicals, to promote the oxygenation of LDL-c and to facilitate lipid peroxidation[38]. Studies have shown increased uric acid levels are associated with atherosclerosis [39]. Apart from the strong correlation with creatinine and eGFR as expected, our data from MetS patients demonstrated that cystatin C was significantly positively correlated with uric acid. These findings are in accordance with previous reports about cystatin C and uric acid in patients with type 2 diabetes [40] and in a hypertensive population [41].

There were some limitations in our study which should be considered. First, we did not collect 24-hour urine for direct measurements of GFR because of its difficulty in daily practice. Instead, we assessed renal function with the commonly used Cockcroft-Gault formula which has some minor flaws that may lead to substantial bias in the assessment. However, numerous studies have demonstrated that the creatinine-based eGFR provides a good approximation. Second, to avoid confounding, we adjusted for well-known confounders such as gender, age, smoking status, and BMI. However, residual confounding may have occurred. Third, our results are derived from a cross-sectional study. Further studies, especially larger, population-based prospective studies are required to validate the findings suggested by the current study.

## Conclusions

In conclusion, our findings demonstrate that cystatin C was significantly associated with the presence and severity of asymptomatic CAD in MetS patients with normal kidney function. Probably cystatin C is more than a marker of glomerular filtration rate. Further research is warranted to clarify the pathophysiologic mechanisms responsible for this association.

## Competing interests

The authors declare that they have no competing interests.

## Authors’ contributions

X Qing, W Xuping participated in the design of the study, W Furong, L Yunxia performed the statistical analysis, X Qing drafted the manuscript. Z Jian, X Qing contributed to the acquisition of data and its interpretation. G Ling, X Qing conceived of the study. All authors read and approved the manuscript.
